# Contrasting Population and Life History Responses of a Young Morph-Pair of European Whitefish to the Invasion of a Specialised Coregonid Competitor, Vendace

**DOI:** 10.1371/journal.pone.0068156

**Published:** 2013-07-03

**Authors:** Odd Terje Sandlund, Karl Øystein Gjelland, Thomas Bøhn, Rune Knudsen, Per-Arne Amundsen

**Affiliations:** 1 Norwegian Institute for Nature Research, Trondheim, Norway; 2 Department of Arctic and Marine Biology, Faculty of Biosciences, Fisheries and Economics, University of Tromsø, Tromsø, Norway; 3 GenØk – Centre for Biosafety, Tromsø, Norway; 4 Institute of Pharmacy, Faculty of Health Sciences, University of Tromsø, Tromsø, Norway; Technical University of Denmark, Denmark

## Abstract

Invasions of non-native species represent a global problem of great scientific interest. Here we study in detail the response in population and life history characteristics of closely related native species, with divergent habitat preferences, that are impacted by an invading species over a sufficient time period to allow a new stable state to become established. A time series of 20 years starting at the first occurrence of the invader (vendace *Coregonus albula* (L.)) allows exploration of the long term population and life history response of two ecologically, morphologically, and genetically different native sympatric morphs (DR- and SR-) of congeneric whitefish *C. lavaretus* (L.). The whitefish morphs are taxonomically equally related to the invading vendace, but only the planktivorous DR-whitefish share its pelagic niche. We would expect that the ecological differences between the whitefish morphs may be used as a predictor of competitive effects. Vendace exhibited an initial boom-and-bust development, and has continued to fluctuate in density. The responses of the pelagic DR-whitefish were: i) an immediate habitat shift, ii) a subsequent population decline caused by increased annual mortality, and iii) a new stable state at a lower density and apparently relaxed competition. The ecologically more distant benthivorous SR-whitefish also showed significant, but a much more limited response during this process, indicating damped indirect interactions through the food-web. This long-term case-study found that in two native eco-species equally related to the invader, only one of the eco-species was highly affected. Direct competition for resources is obviously important for species interactions, whereas the taxonomic relatedness *per se* seems to offer little predictive power for invasion effects.

## Introduction

The invasion and establishment of non-native species may cause serious disruption in the recipient ecosystem (e.g. [1], [2]), and may in some cases be termed an event tipping the system into a new stable state (cf. [3]). The invading species (or invader) may constitute a serious competitor for food and habitat, and/or it may become a predator or prey affecting the native species populations. The naturalization hypothesis suggests that competitive effects are stronger between closely related (i.e. phylogenetically similar) species than between less similar species [4], [5], [6]. Few studies have, however, addressed in detail the response in population and life history characteristics of phylogenetically closely related native species that are impacted by an invading species over a sufficient time period to allow a new stable state to become established.

In many cases, the invading species have been shown to go through a so called “boom-and-bust” cycle, with apparently dramatic changes during the “boom”-phase (i.e. with swiftly increasing population densities), followed by a decline (“bust”) and subsequent stabilization at a moderate or even low population density [7], [8], [9]. The duration of this transient boom-and-bust phase and stabilization into a new long-term equilibrium state of the ecosystem, including data on the characteristics and life history traits of its affected populations, have to our knowledge not been documented.

The impact of an introduced species in an ecosystem is primarily associated with two dimensions, i) the amount of resources *per capita* or biomass consumed by, and ii) the abundance of, the introduced species (cf. [10]). To become abundant and achieve any significant impact, i.e. to be termed an invasive or invading species (cf. [11]), the introduced species must compete with native species for habitat and resources. By usurping resources which previously were utilized by native species, the invasive species has an impact on the community [12]. The competitive characteristics of the introduced species relative to native species in the recipient ecosystem, is a crucial factor for the interactions and thus for determining the impact of the invasion [13]. It may further be expected that native species which are ecologically similar to the invasive species might be less resilient in facing the potentially increased competition after the establishment of the new species [6], [14], [15]. Phylogenetic distance between the native and introduced non-native species may also influence the outcome [5]. Finally, plasticity in resource requirements of the native and/or the non-native species may determine whether they are able to co-exist [16].

In order to understand the process of introduction and invasion, via establishment and early population development through to the stabilized new situation, it is necessary to follow the non-native species as well as the recipient ecosystem for a prolonged period of time. The invasion process may include at least three stages: the introduction itself, the establishment of a population of the non-native species, and the subsequent population development of the non-native species. The non-native species population may remain at a low density with very little detectable impact, or it may turn out to be invasive (cf. [11]), increasing in population density with substantial impact on native species. To understand the long-term impact, it is necessary to follow the non-native invasive species and the native species through several generations until some alternative state of the ecosystem is established.

Invasions of fish species in aquatic ecosystems are often investigated after the fact, as the occurrence of the non-native invasive species is commonly recorded only when it has developed a well-established population. In the case of the vendace (*Coregonus albula* (L.)) invasion in the Pasvik watercourse in Northern Fennoscandia, however, we have been able to follow the vendace and the recipient ecosystem annually from the initial stages of invasion in 1991 [17]. The development of the invading population as well as the populations of two native sympatric morphs of whitefish (*C. lavaretus* (L.)) has been recorded through a time period of 20 years covering 3–10 generations of both whitefish and vendace. The native whitefish morphs are the planktivorous densely-rakered (DR-) whitefish, originally associated with the pelagic zone, and the benthivorous sparsely-rakered (SR-) whitefish feeding in benthic habitats [17], [18], [19]. Although they have the same taxonomic nomenclature, DR- and SR-whitefish are morphologically, ecologically and genetically separated. The whitefish morphs have apparently developed in sympatry [20], and their phylogenetic distance to vendace is therefore identical. In ecological terms, however, DR-whitefish is nearly identical to vendace, whereas SR-whitefish has longer generation time and a different ecology in terms of habitat and diet [18]. Functionally, vendace and the two whitefish morphs can therefore be treated as three separate eco-species.

In this geographic region (northern Fennoscandia) there are numerous lakes with similar dimorphic whitefish populations where vendace has not been introduced [21]. Several of these lakes have been investigated for other purposes, but the results strongly indicate that the dimorphic population structure is stable over time [22], also in the face of significant population reductions due to exploitation [23].

In this paper we investigate the changes in population parameters in the three coregonid eco-species over the initial two decades following the invasion of vendace. The development of life history parameters in vendace during the first ten years of the invasion has been analysed earlier [24]. With 20 years of data we investigate if a new stable state has been reached, and we focus in particular on the native zooplanktivorous DR-whitefish. First, we would expect that the population parameters and life history traits of DR-whitefish will be shifted away from those of vendace as a consequence of strong competition and the initial overlap with vendace in trophic niche. Second, we hypothesise that SR-whitefish should be less impacted in population density and life history traits because of low trophic niche overlap with vendace and the DR-morph. Finally, as vendace have been part of the fish community for 20 years, covering multiple generations in the three eco-species, we would expect a stabilization of population densities and life history parameters in a new equilibrium state. This overall process of the invasion, and its consequences, are evaluated by analysing the development of population and life history parameters of the three coregonid eco-species over the time period since the ecosystem was invaded by vendace.

## Materials and Methods

### Study Area

The Pasvik watercourse (69°N, 29°E) originates in Lake Inari (1,102 km^2^) in Finland, runs into Russia and then forms the border between Norway and Russia over a distance of about 120 km. The Norwegian-Russian part of the watercourse has a total area of 142 km^2^, a catchment area of 18,404 km^2^ and a mean annual water flow of about 175 m^3^ s^−1^. There are a total of seven water impoundments (hydropower reservoirs) in this part of the watercourse. Most rapids and waterfalls have disappeared and the former river system now primarily consists of lakes and reservoirs linked by slow-flowing river sections. The water level fluctuations in the study lake are small, usually less than 80 cm, and the ice-free season lasts from May – June to October – November.

The lakes and reservoirs are oligotrophic with relatively humic waters, with low concentrations of total phosphorous and nitrogen (Table 1). The catchment area is thinly populated, with less than 1000 people in the Norwegian section of the Pasvik valley, which constitutes more than 1300 km^2^. The present study was carried out in southern Lake Vaggatem (69°13′ N, 29°14′ E), consisting of two basins, Ruskebukta and Tjærebukta, located in the upper part of the Pasvik watercourse (Table 1). Along the lake there are only a few scattered farms, mainly producing grass for dairy cattle. A total of 15 fish species have been recorded in the Pasvik watercourse, but in Lake Vaggatem the most common in addition to the coregonids are: Eurasian perch (*Perca fluviatilis* L.), northern pike (*Esox lucius* L.), burbot (*Lota lota* (L.)), 9-spined sticklebacks (*Pungitius pungitius* (L.)) and brown trout (*Salmo trutta* L.).

**Table 1:** Locality characteristics of southern Lake Vaggatem.

Altitude	52 m
Surface area	15 km^2^
Maximum depth	30 m
Ice cover	Nov.-May
Secchi depth	1–4.5 m
pH	6.8
Total P	9 µg L^−1^
Total N	145 µg L^−1^

Vendace did not occur in the Pasvik catchment until the 1950s, when it was translocated from southern Finland to tributaries to Lake Inari. The first vendace was caught in Lake Inari in 1973 [25], and by the end of the 1980s the lake had a very large and commercially exploited vendace population [26]. Downstream migration with resulting establishment of populations in the Pasvik river system appears to have commenced at this time. No vendace was caught during survey net fishing in 1982 in several Pasvik lakes, including Lake Vaggatem [27]. Vendace was caught for the first time in the Pasvik watercourse by a local commercial fisherman in 1989 (G. Kalliainen, pers. comm.). A small coregonid fish sample purchased for a different research project from Mr. Kalliainen in 1990 contained a few vendace (9 out of 120 fish; O.T. Sandlund, unpublished data), and was the first vendace observation verified by a researcher. Both the 1989 and 1990 observations were made in a hydropower reservoir upstream to the present study lake. Thus, our first population investigation in 1991 sampled the vendace invasion in its early stage.

### Fish Sampling

Fish sampling was performed annually from 1991 to 2010 (except 1994 and 1996) in the period 10–21 September (Table 2). Fish sampling was performed both in the littoral (<8 m), profundal (>10 m) and pelagic habitats using gillnets during night-time. The gillnets were 40 m long containing eight sections of 5 m with different mesh sizes. In the pelagic zone, 6 m deep floating nets were used, whereas 1.5 m deep bottom nets were employed in the littoral and profundal zones. The mesh sizes used were 8, 10, 12.5, 15, 18.5, 22, 26, 35 and 45 mm (knot to knot) in the pelagic habitat, and the 10–45 mm range in the benthic habitats. As a response to reduced vendace growth and smaller 0+ lengths, 6 mm mesh size was also included in the pelagic gill nets from 2002. Catch per unit effort (CPUE) was used as an indicator of fish density, excluding 0+ from all years due to the variable gillnet catchability of this age class. The profundal zone is small compared to the total lake surface [28]. Consequently, as an approximation in order to obtain a total CPUE as an overall indicator of fish abundance, habitat-specific CPUE values were averaged weighting the littoral, pelagic and profundal habitats by 1, 1, and 0.5, respectively. CPUE was calculated and presented in terms of numbers per net area and time (CPUE_numbers_, # fish • 100 m^−2^ • 12 hrs^−1^), and as fish mass per net area and time (CPUE_weight_, g fish • 100 m^−2^ • 12 hrs^−1^).

**Table 2:** Number of fish collected in southern Lake Vaggatem, 1991–2010.

Year	DR-whitefish	SR-whitefish	Vendace	Total
1991	147	35	70	253
1992	183	21	36	240
1993	242	46	496	790
1995	213	70	280	579
1997	97	37	207	347
1998	68	43	480	595
1999	33	4	166	203
2000	47	14	155	216
2001	64	31	135	232
2002	81	41	245	367
2003	53	37	47	159
2004	110	78	667	862
2005	59	126	283	490
2006	44	40	129	242
2007	153	96	141	393
2008	83	93	174	351
2009	109	82	696	901
2010	112	95	625	835
Grand Total	1898	989	5032	8055

All fish were measured in mm (fork length) and weighed in grams. Fish age was determined from surface readings of otoliths and counting the number of hyaline winter zones. The sex and immature or mature status were determined. As the fish were caught shortly before spawning (which is in October), this is diagnostic. For a sub-sample of fish collected in some of the years (1995–2010 for vendace, 1998, 2007, and 2010 for both whitefish eco-species), female gonads were weighed in milligrams and thereafter stored on Gilson’s solution. In the lab, a subsample of 50–150 eggs from each gonad was weighed and the number of eggs counted, and the diameter of 15 eggs were measured. The total number of eggs in the gonad was estimated by multiplying the number of eggs in the subsample by the ratio of total egg mass to subsample egg mass. The gonadosomatic index *GSI* (%) was calculated according to the formula *GSI* = 100 · *M_G/_*(*M_T_ - M_G_*), where *M_T_* is total fish weight and *M_G_* is gonad weight. As data on female gonads is available only for some years for the whitefish eco-species, the parameters egg number and GSI are used to indicate differences among the eco-species on a general level only.

According to regulation FOR 1996–01–15 no. 23 on experiments with animals (Ministry of Agriculture and Food) no ethical permit is required for collection with gill nets and the associated killing of fish. A fishing permission is required from the fishing right owner, which on Government land in Finnmark is the County Governor of Finnmark. We obtained annual permissions for the gill net fishing in Lake Vaggatem from the County Governor.

### Statistical Analysis

To model population trends and interaction strengths we used state-space models (developed in the R software) accounting for both process noise and observation errors. The models are also capable of handling missing data. This is a limitation in many other methods for time series analysis. The population trends (abundance) for each of the populations were estimated using an exponential growth state-space (EGSS) model. Two approaches were applied with this method; first we modelled the full time series from 1991 to 2010, thereafter we modelled the invasion phase (1991–1999) and post-invasion phase (2000–2010) separately. The rationale for this division was that the invasion phase may be regarded as a strong system perturbation, whereas in the post-invasion phase the system has had more time to stabilize (i.e. the process changes from non-stationary to stationary). We used the EGSS method described by Humbert et al. [29]. This method models population growth using an exponential growth model without density dependence or species interactions (Eqn 1).
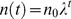
(1)Where n(t) and n_0_ are the population abundances at time t and zero, respectively, and is the population growth rate.

A general stochastic version of this exponential growth model including both process noise and observation error has an unobserved population component and a component representing the observed abundance values (Eqn 2).
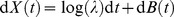
(2)

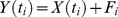



Here, *X*(t) is the unobserved log-abundance of the population at time t and *Y*(t) the observed value of *X*(t). The term d*B*(t) (∼normal(0,σ^2^)) is a random perturbation representing the process noise (environmental variability), and *F*_i_ (∼normal(0,τ^2^) represents the observation error, assumed to have no auto- or cross-correlations. Log(λ) is the expected change of *X*(t) in one time unit, and serve as our trend parameter. Further details of this EGSS model can be found in Humbert et al. [29].

We used multivariate autoregressive state-space modelling (MARSS) to estimate species interactions from the CPUE data. A MARSS model, with Gaussian errors, takes the following form [30], [31]:
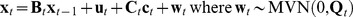
(3.1)

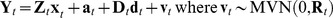
(3.2)

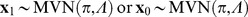
(3.3)


The **x** equation is termed the state process and the **y** equation is termed the observation process. Data enter the model as the **y**; that is the **y** is treated as the data although there may be missing data. The c*_t_* and d*_t_* are exogenous inputs (e.g. covariates or indicator variables). The bolded terms are matrices with the following definitions: **x** is a *m*×*T* matrix of states. Each **x** *_t_* is a realization of the random variable **X** *_t_* at time *t*. **w** is a *m*×*T* matrix of the process errors, the process errors at time *t* are multivariate normal (MVN) with mean 0 and covariance matrix **Q** *_t_*. **y** is a *n*×*T* matrix of the observations. **v** is a *n*×*T* column vector of the non-process errors (observation errors), the observation errors at time *t* are multivariate normal with mean 0 and covariance matrix **R** *_t_*. **B** *_t_* and **Z** *_t_* are parameters and are *m*×*m* and *n*×*m* matrices. **u** *_t_* and **a** *_t_* are parameters and are *m*×1 and *n*×1 column vectors. **Q** *_t_* and **R** *_t_* are parameters and are *m*×*m* and *n*×*n* variance-covariance matrices. **π** is either a parameter or a fixed prior, it is a *m*×1 matrix. **Λ** is either a parameter or a fixed prior, it is a *m*×*m* variance-covariance matrix. **C** *_t_* and **D** *_t_* are parameters and are *m*×*p* and *n*×*q* matrices. **c** and **d** are inputs (no missing values) and are *p*×*T* and *q*×*T* matrices.

The modelling was performed with the R-package “MARSS” [30], [31], using the following formula for the z-transformed log abundances *x*:
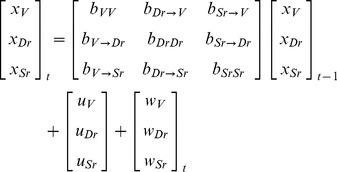
(4)

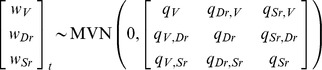



Symbols in Eqn 4 are as for Eqn 3, with the subscripts *V*, *Dr*, and *Sr* for vendace, DR-whitefish and SR-whitefish, respectively. The **B** matrix (*b*-parameters) is the interaction terms between the eco-species, with the diagonal giving the intraspecific interactions (density dependence. We subtracted the **B** diagonal by 1 in order to have the same interpretation of diagonals as off-diagonals [31]). In the MARSS package, parameters can either be estimated, or they can be specified. We specified the observation variance as 0.23 for all ecospecies, the mean of within year gillnet CPUE variances. First, all species interactions were estimated as the initial model (full model). Then we removed interaction terms (by specifying them as 0) in a stepwise procedure, removing parameters with estimated values closest to zero. The performance of the reduced model was evaluated by the AIC (Akaike Information Criterion) value, and the reduced model was kept as long as the reduced model AIC were lower than the AIC from the previous model step. The 95% confidence intervals were also evaluated in order to check if interaction terms significantly different from zero were dropped from the model. Running the MARSS model for only invasion phase data did not reach convergence, hence MARSS modelling is only presented for the full study period from 1991–2010.

We used logistic regression [32] with immature and mature fish as the binomial response variable to estimate the age and length at which 50% of the fish were sexually mature. Due to low sample numbers, for some of the analyses it was necessary to compile data into six consecutive time periods, including 1991–1993 (Period 1), 1995–1997 (Period 2), 1998–2000 (Period 3), 2001–2003 (Period 4), 2004–2006 (Period 5) and 2007–2009 (Period 6). Similarly, compiled data for the six time periods were used to estimate instantaneous mortality rates (Z), using standard age-sorted catch curves from the fish surveys (see [33]), omitting fish <1 year old from the estimations due to biased selection of the smallest fish. The age distributions were pooled for all years within each time period in order to reduce the effect of year class variation [33], which often is pronounced in vendace populations [34], [35], [36]. The annual mortality rate (A) was calculated as *A = 1 - e* ^−*Z*^.

In order to compare the somatic growth rate of vendace between time periods, the growth (length at age) was modelled for each cohort from 1990 to 2008, using the von Bertalanffy growth model [37]:
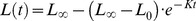
(5)where *L(t)* is the mean fish length at age *t* (age as number of growth seasons, i.e. young of the year in September will be age 1 in this analysis). *L*_∞_ is the mean asymptotic length as age approaches infinity, *L_0_* is the length at hatching (age 0), and *K* (Brody’s growth coefficient) defines the rate at which the growth curve approaches the asymptote. However, the unit-less *K* is strongly dependent on, and cannot be understood without, knowledge of *L*_∞_. In order to obtain a growth measure that is less dependent on *L*_∞_ and may be interpreted across populations and years, we estimated absolute initial growth rate (*G_i_*) by a modified version of the von Bertalanffy equation [38], [39]:



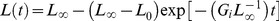
(6)
*G_i_* is also easier to interpret in terms of growth, since it has the unit length • age^−1^ and represent the maximum growth rate, occurring early in life according to the von Bertalanffy growth model. *L*_∞_ and *G_i_* were estimated by non-linear least-square regression based on mean lengths of each age-class. *L_0_* were set to 11 mm for vendace and 16 mm for DR- and SR-whitefish [40], [41].

Fish condition was calculated as Fulton’s condition factor *K_c_* [33]:
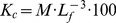
(7)where *M* is the fish body mass (g) and *L_f_* is the fork length (cm). For all eco-species, there was a significant, positive and linear relationship between *L_f_* and *K_c_*. Residuals from the linear regression models were compared between eco-species by Pearson correlation coefficient in order to evaluate between-year co-variation in fish condition, and to evaluate whether the invader (vendace) CPUE had any influence on the condition of the native coregonid eco-species.

In order to investigate for temperature trends, we used a time series of daily mean water temperatures from a temperature logger located at 3 m depth at the water intake to the hydropower station downstream of Lake Vaggatem (Skogfoss hydropower facility operated by Pasvik Kraft AS, data owner Norwegian Water Resources and Energy Directorate). The temperature time series was statistically treated by a generalized additive mixed model [42], using year and day of year as fixed effects. The day of year term effect was modelled using penalized regression smooth spline [42], as daily temperature is an inherent non-linear function of the time of the year. The year effect was also modelled using penalized regression smooth spline in one regression model, and as a linear effect in an alternative model. These models were subsequently compared, and the model with a linear year effect was chosen based on a lower AIC value. Autocorrelation effects were removed by including a first-order autoregressive model nested by year in the model specification.

## Results

**Figure 1:**
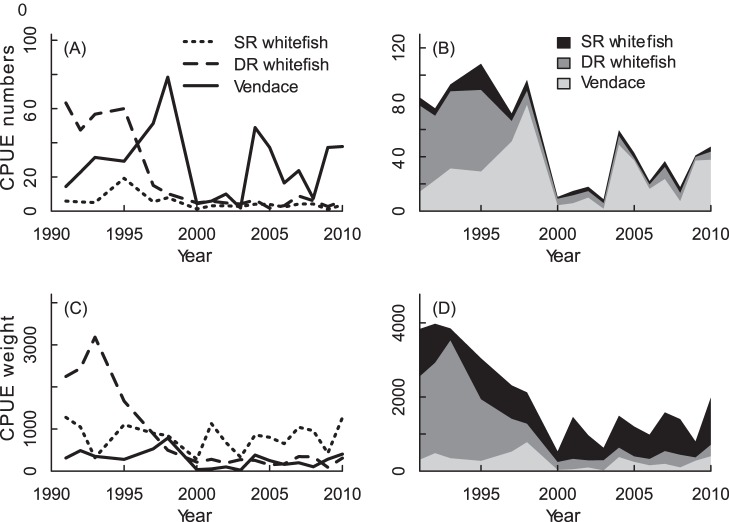
Gillnet catch per unit effort (CPUE) of vendace, densely-rakered (DR) whitefish and sparsely-rakered (SR) whitefish from 1991 to 2010. A and B: CPUE by number of fish per 100 m^2^ gill net area. C and D: CPUE by weight, g fish per 100 m^2^ gill net area. Lines in A and C show species-specific CPUE averaged over the littoral, pelagic and profundal zones (weighted by 1, 1, and 0.5, respectively), whereas B and D show the cumulative CPUE for the averages in A and C, respectively.

The time series of gillnet catches (CPUE) from 1991 to 2010 indicates that CPUE was at a relatively high level before 2000, while in later years total catches seem to have stabilized at a somewhat lower level (Figure 1 A, B).

Considering the catches of the three eco-species individually, vendace exhibited a marked increase in terms of numbers from 1991 until 1998 (Figure 1 A). The increase in terms of weight was much less (Figure 1 C), reflecting the decreasing vendace mean body size. The maximum population density of vendace in 1998, with a CPUE of 78.4 fish, was followed by a swift decline to a CPUE of 4.4 fish in 2000. Remaining at a low level until 2003, vendace CPUE again increased to 48.9 fish in 2004. In recent years, vendace catches have varied greatly, with CPUE values between 7.4 and 37.3 fish. The population crash of vendace in 2000 was also reflected in terms of biomass (Figure 1 C), although with its small individual body mass, vendace had less impact on the total CPUE in terms of biomass (Figure 1D).

The catches of DR-whitefish in number of fish, in contrast, remained relatively high during 1991–95 (CPUE = 47.4–63.3), but with a subsequent dramatic decrease to a CPUE of 4.8 fish in 2000 (Figure 1 A). In later years, catches of DR-whitefish have remained relatively stable at a low level (CPUE = 1.7–8.8). In terms of biomass, CPUE of DR-whitefish was at a maximum in 1993 (2652 g), while since 2000, it has varied between 132 and 278 g (Figure 1 C, D). When DR-whitefish had reached a lower level of abundance (2000–2010; cf. Figure 1), there were only minor changes in catches over time.

SR-whitefish has shown a less dramatic change after the introduction of vendace (Figure 1 A, B). Catches have been decreasing slightly both in terms of numbers and weight, and fluctuations were less prominent than for the other two eco-species. Due to its larger individual body mass, SR-whitefish has throughout the period made up a substantial proportion of the catches in terms of biomass (Figure 1D).

The distribution of DR-whitefish catches in number of fish varied between the pelagic and epibenthic habitats, but there was a very clear difference between the (early) invasion phase and later years. From 1991 through 1993, pelagic CPUE of DR-whitefish decreased from 127 to 10 fish, while over the same period, epibenthic CPUE increased from 32 to 132 fish. In 1995, there was a rebound to higher pelagic and lower epibenthic catches, with 96 and 54 fish, respectively. Since 1997, however, pelagic CPUE of DR-whitefish has remained at between zero and 16 fish. Epibenthic CPUE decreased to 23 fish in 1997, and has remained between 1 and 15 fish since 1998.

**Figure 2:**
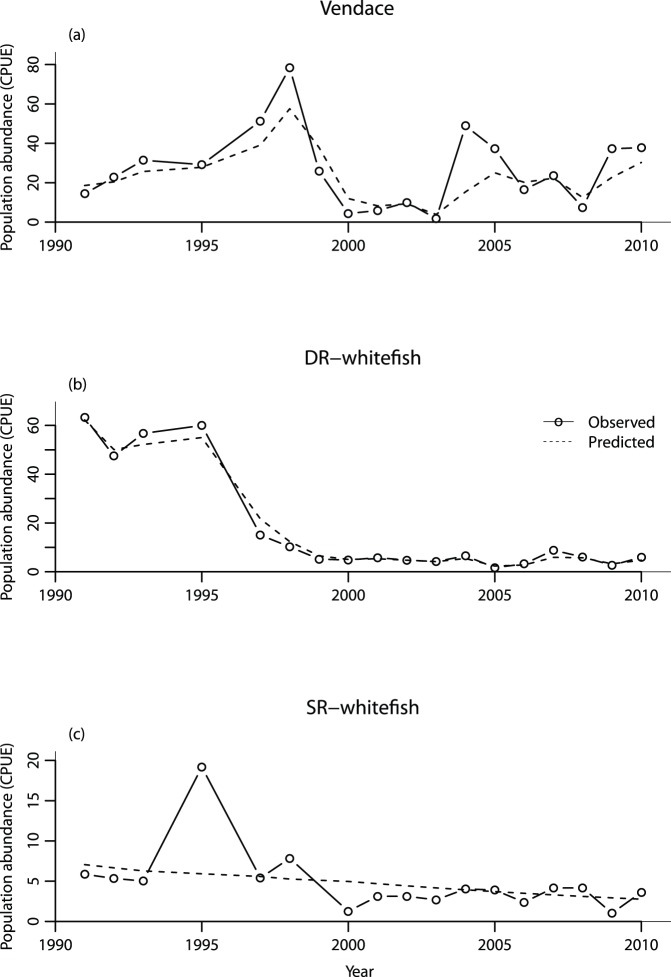
Population trends as observed (circles connected by solid lines) and as estimated with the EGSS modelling procedure for the full 1991–2010 period (dashed lines) for vendace (A), DR-whitefish (B), and SR-whitefish (C).

The population trend analyses by EGSS-modelling indicated overall negative trends of SR-whitefish for the period 1991–2010, while for vendace and DR-whitefish, the trends were not different from zero over this period (Table 3, Figure 2). However, during the invasion phase (1991–1999), vendace had a significant population increase while DR-whitefish had a significant population decrease (Table 3). After the population crash between 1998 and 2000, vendace again had a significant population increase from 2000 to 2010. During this period, the population trend of DR-whitefish was not significant from zero (Table 3), reflecting an apparently stable population at a low level (Figure 2).

**Table 3:** Results from the exponential growth state-space (EGSS) population trend analyses.

Eco-species	Period	Log(λ)	Lower 95% C.I.	Upper 95% C.I.
Vendace	1991–1999	**0.12**	0.008	0.24
	2000–2010	**0.19**	0.024	0.36
	1991–2010	0.026	−0.26	0.31
DR-whitefish	1991–1999	−**0.31**	−0.61	−0.019
	2000–2010	−0.005	−0.10	0.087
	1991–2010	−0.14	−0.33	0.057
SR-whitefish	1991–1999	0.042	−0.46	0.54
	2000–2010	0.024	−0.12	0.17
	1991–2010	−**0.059**	−0.11	−0.012

**Figure 3:**
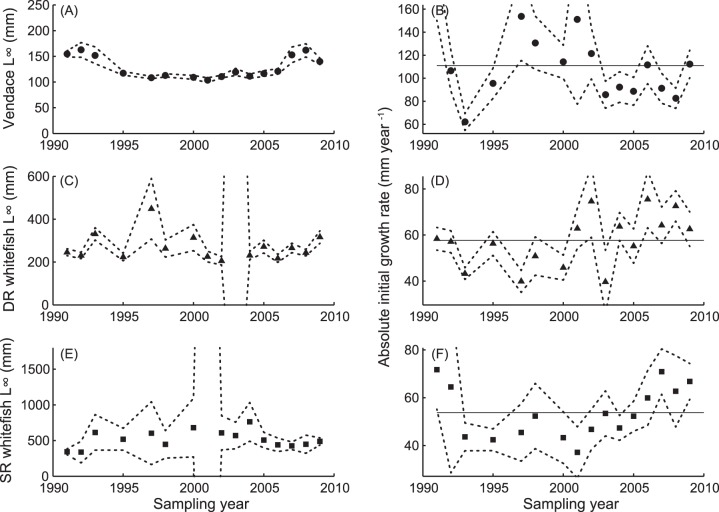
Parameters from the von Bertalanffy growth models, based on the age and length structure observed in yearly catches of vendace (A, B), densely-rakered (DR) whitefish (C, D), and sparsely-rakered (SR) whitefish (E, F). The changes in *L_∞_* with time are shown in the left panels (A, C, E). In the right panels (B, D, F) the development in absolute initial growth rate is shown, with the mean indicated by the solid horizontal line. The dotted lines indicate parameter 95% confidence intervals.

Positive log(λ) indicates increasing population trend, negative log(λ) indicates decreasing population trend. Log(λ) in bold characters indicates values significantly different from zero as determined by the confidence intervals (C.I.). Results from the full period analyses (1991–2010) are shown in Fig. 3.

The initial results from the MARSS time series modelling revealed the interaction terms shown in Table 4. In the full model, the three strongest effects were the negative effects of vendace on all three eco-species. However, in the final model based on the model selection criteria, only density dependence in vendace and DR-whitefish were significant effects, as indicated by parameter estimates different from zero (Table 4).

**Table 4:** Matrices of parameter estimates from the multivariate autoregressive state-space (MARSS) modelling procedure.

Model		Vendace	DR-whitefish	SR-whitefish
Full model	Vendace	−0.71	0.217	0.058
AIC*: 131.0	DR-whitefish	−0.23	−0.108	0.167
	SR-whitefish	−0.28	0.047	0.079
Final model	Vendace	−0.69 (−1.1, −0.26)	0	0
AIC*: 124.2	DR-whitefish	0	−0.15 (−0.23, −0.06)	0
	SR-whitefish	0	0	0

Parameter matrix diagonals have been subtracted by 1 to obtain the same parameter interpretation for diagonals and off-diagonals [47]. The matrix diagonals show intra-population density dependence, whereas the off-diagonals show interaction terms between the eco-species. Negative values indicate negative density dependence/negative interspecific interactions. Parameter_i,j_ shows the influence of eco-species j on eco-species i, where i is the row number and j is the column number. Parameter 95% confidence intervals are given (lower, upper) for the final model. Parameter value 0 indicates that the interaction was removed from the model by setting the parameter to 0.

* AIC: Akaike information criterion.

The condition factor model residuals were positively correlated between vendace and DR-whitefish (r_Pearson_ = 0.55, P = 0.011), and between vendace and SR-whitefish (r_Pearson_ = 0.55, P = 0.012). There were no significant correlations between vendace pelagic CPUE and concurrent condition factor residuals for any of the species. However, there was a significant negative correlation between vendace pelagic CPUE and condition factor residuals in DR-whitefish one year later (r_Pearson_ = 0.51, P = 0.026), a nearly significant negative correlation between vendace CPUE and vendace condition factor residuals the year after (r_Pearson_ = 0.44, P = 0.060), but no correlation between vendace CPUE and SR whitefish condition factor residuals (r_Pearson_ = 0.12, P = 0.81). These results indicate that community responses to competition were mainly in terms of reduced fish abundance, but in part also in next year’s condition factor.

Growth parameters (according to the von Bertalanffy growth model) varied throughout the sampling period in all three eco-species (Figure 3). Vendace L_∞_ was high during 1991–1993, decreasing to a lower level which was maintained from 1995 to 2005. Thereafter, L_∞_ increased to a new peak in 2008. The G_i_ (absolute initial growth rate) for vendace has oscillated greatly over time, but has stabilized at a lower level during 2003–2009 than during 1997–2002 (Figure 3). No clear trend in L_∞_ was observed for DR- and SR-whitefish. Both whitefish eco-species experienced a rapid decrease in G_i_ shortly after the vendace appearance, but after a period of stable values below the mean, G_i_ has again increased during the last decade (Figure 3). It may appear that the G_i_ values of DR- and SR-whitefish remained low while vendace G_i_ was high, and vice versa. These patterns are supported by the 95% parameter confidence intervals.

**Figure 4:**
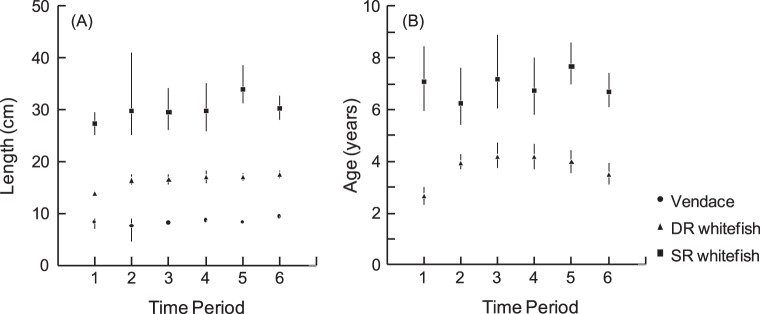
Mean size (A) and mean age (B) at first reproduction (i.e., body length and age with 50% mature individuals) estimated by logistic regression. (Period 1 = 1991–1993, 2 = 1995–1997, 3 = 1998–2000, 4 = 2001–2003, 5 = 2004–2006, 6 = 2007–2009). Error bars show 95% confidence intervals. For vendace, >50% of the individuals were mature at age 1 in all time periods.

The difference between vendace and DR-whitefish in mean length at first maturity was 57 mm in 1991–93 (Figure 4). In 1995–97, DR-whitefish increased in size at first maturity while vendace remained at almost the same length. The increased difference in adult body size between the two zooplanktivorous eco-species was maintained in later sampling periods including 2007–09. In SR-whitefish, mean length at first maturity remained around 300 mm, but with considerable variation around the mean. Considering age at first maturity (Figure 4B), DR- and SR-whitefish varied considerably throughout our sampling period, whereas more than 50% of vendace matured at age 1+ (i.e. the second growth season) in all sampling periods. Age at first maturity in DR-whitefish increased from 2.6 years in 1991–93 to 3.9 years in 1995–97. In later periods, maturation age was relatively stable, but with a weakly decreasing trend from 2001–03 to 2007–09.

**Figure 5:**
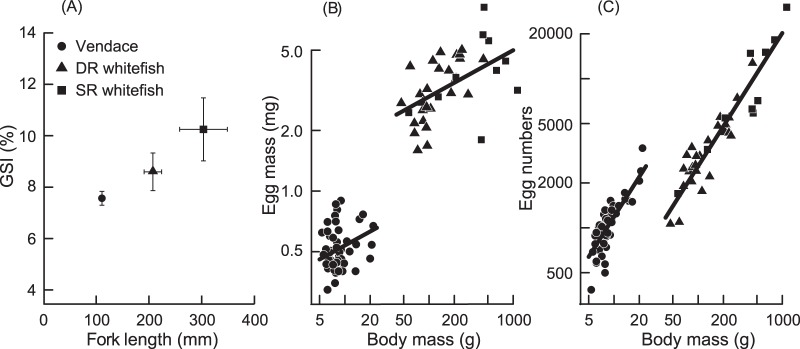
Gonadosomatic index (GSI) and mean body length of mature females, and egg mass and egg numbers vs. body mass of the three coregonid eco-species. A: Mean fish length and gonadosomatic index (GSI), with error bars showing 1 SE. DR = densely-rakered whitefish, SR = sparsely-rakered whitefish. The difference in GSI was significant between all three ecospecies (p<0.001). B: Egg mass vs. body mass. Mean egg mass per female fish was modelled with the statistical model = log(Egg mass) x Ecospecies, model predictions indicated by lines. Confer text for statistical results. C: Egg numbers vs. body mass.

To compare life history strategies among the three ecospecies, we have estimated the gonadosomatic index (GSI) based on a subsample of fish. GSI differed among the three coregonid eco-species (Figure 5). The relative female investment in gonads was significantly higher in SR-whitefish compared with DR-whitefish (P = 0.005) and vendace (P<0.001), and higher in DR-whitefish than in vendace (P = 0.015). The mean single egg mass increased with fish body mass (model = log(Egg mass) x Eco-species, interaction term (P = 0.30) was removed, P_egg mass_<0.001), and was larger in the whitefish morphs than in vendace (P<0.001). The egg mass also differed between whitefish morphs, but was largely explained by differences in fish size. The slope of the relationship between egg mass and body mass did not differ significantly between vendace and the two whitefish morphs, and the slope for vendace was not significantly different from zero. The individual fecundity was strongly correlated to body mass for all eco-species (P<0.001). The fecundity was much lower in the two whitefish morphs than in vendace given a similar size (P<0.001), but no significant difference was found between the two whitefish morphs (P = 0.89). There was no evidence for a different slope between the eco-species (P = 0.30).

**Figure 6:**
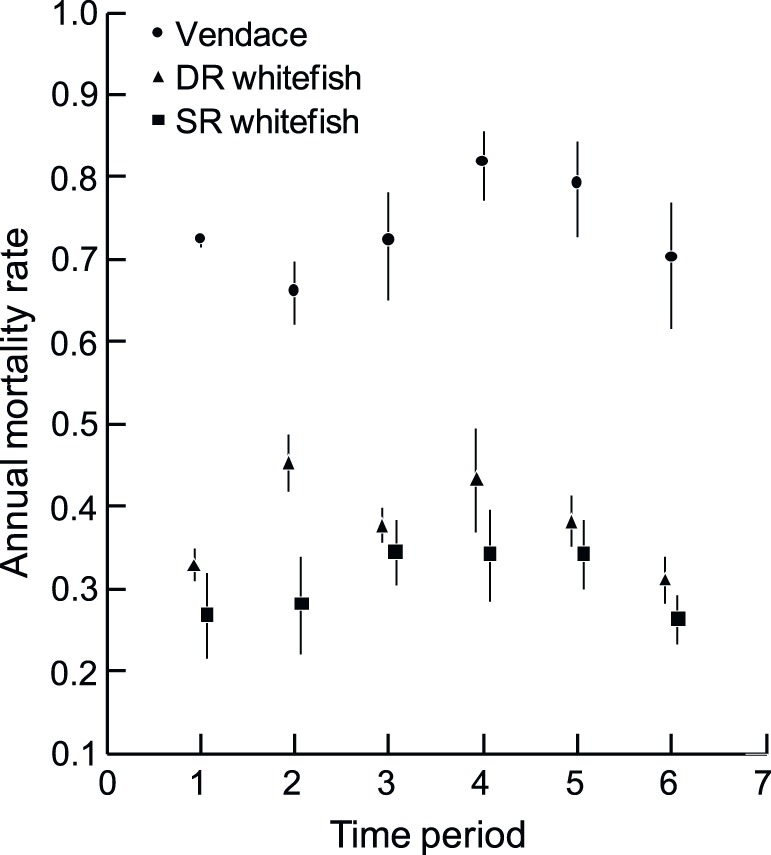
Annual mortality rates for vendace, densely-rakered (DR) whitefish and sparsely-rakered (SR) whitefish. Error bars are 1 SE. (Period 1 = 1991–1993, 2 = 1995–1997, 3 = 1998–2000, 4 = 2001–2003, 5 = 2004–2006, 6 = 2007–2009).

The annual mortality was similar for DR- and SR-whitefish during the early phase of the vendace invasion (1991–93), but increased substantially for DR-whitefish during the main boom-period of vendace (1995–97) and remained relatively high until 2004–06 (Figure 6). The mortality of SR-whitefish was high only during the period 1998–2006. The annual mortalities of DR- and SR-whitefish morphs were back on a level similar to that observed during the early invasion phase 16–18 years after the vendace invasion (i.e. the 2007–09 period).

The statistical model of the daily mean water temperatures from 1991–2010 gave a good fit to the data (r^2^ = 0.97) and revealed a slightly positive temperature trend over years (an increase of 0.017°C year^−1^), but this effect was not significant (P = 0.31).

## Discussion

Two decades with annual data on the invasion process indicate that many population parameters of the non-native invasive species (the invader) as well as the native whitefish populations were strongly affected during the dynamic invasion phase. However, after a transitional period that lasted about 10–12 years, corresponding to 6, 4, and 2 generations of vendace, DR- and SR-whitefish, respectively, a new stable state seems to have been reached.

After the introduction of vendace in Lake Vaggatem, the coregonid fish community has followed a trajectory from its native undisturbed structure through invader establishment, and a transient period, which included an invader boom-and-bust phase associated with a strong response, particularly in the ecologically most similar native eco-species. The system seems to have reached a new stable state in the recent years. We will use the term invasion phase for the boom-period, when the invader population density increases to levels which bring changes to the fish community. The period after the invader population has busted is termed the post-invasion phase, characterized by an abundant invader population dominating the pelagic habitat, which previously was dominated by the native zooplanktivorous DR-whitefish. Thus, DR-whitefish has experienced a substantial and significant decline in numbers as well as a shift away from the preferred pelagic habitat to the epibenthic zone. Along with this, a decrease in absolute initial growth rate was observed. In the post-invasion phase, the population abundance of DR-whitefish has stabilized at a substantially lower level than before the invasion, while most life history parameters of this eco-species have returned to values similar to the pre-invasion level. The benthivorous SR-whitefish experienced a reduction in absolute initial growth rate during the invasion phase. Although the relative reduction in density was less than for DR-whitefish, SR-whitefish had a significantly negative population growth rate throughout the study period. This indicates that SR-whitefish also was negatively affected, directly or indirectly, by the vendace invasion. The mortality rates for SR-whitefish were less affected by the invasion than for DR-whitefish.

To conclusively demonstrate the impact of some environmental perturbation on an ecosystem or on species populations, a proper BACI-design (Before-After-Control-Impact, cf. [43]) of the investigation would normally be required. This is often not possible, as the perturbation happens outside the control of the investigator [44]. Data series obtained from monitoring programmes may detect some change in the monitored populations or ecosystems, but most often data from control systems are lacking. Data on relevant environmental parameters may also be lacking, due to budgetary and logistic constraints, as well as to a deficient understanding of the system under scrutiny when the monitoring programme was initiated (cf. [45]). Separation of the effects of different environmental changes may actually be particularly difficult in long-term data series [46], as several environmental factors may vary over time and confound our attempted cause-effect analysis. Our data series from Lake Vaggatem demonstrates changes in the two native whitefish eco-species. We argue that this is due to the impact of the development of the invasive vendace population, representing the major environmental change in this system. From reviewing other environmental factors, we argue by causal inference (cf. [47]) that no other environmental perturbation has been likely to produce the observed change in the whitefish populations in Lake Vaggatem. Furthermore, as it was not possible to apply a proper BACI-design in this case, we will by way of control refer to data on the temporal development of similar dimorphic whitefish populations in other lakes in the region, where no invasion of non-native species has occurred.

Four environmental factors might be envisaged as potential pressures which may bring about substantial changes in the fish populations in Lake Vaggatem. These are temperature changes due to climate warming, eutrophication due to runoff from the catchment area, changes in the operational patterns of the lake as a hydropower reservoir, and pollution from heavy industries in Russia.

The water temperatures recorded at the lake water intake to the hydropower station downstream of Lake Vaggatem from 1991 to 2010 showed no significant change. Neither were there any years with unusually high or low water temperatures during this period.

The Pasvik valley is very sparsely populated with negligible agricultural activity and no urban centers. There has been no noticeable change in human activities in the area, and no change in runoff from the restricted areas of cultivated land. Thus, one may not expect any noticeable change in water productivity away from the oligotrophic situation in the lake. Furthermore, although the lower part of the Pasvik watercourse is under the influence of heavy metal emissions from nearby Russian metallurgic industry [48], no impacts have been revealed for fish in Lake Vaggatem, which is situated 40 km upstream from the smelters [49], [50].

The hydropower concession for Lake Vaggatem stipulates that water level fluctuations of the hydropower reservoir should be kept within 80 cm. There has been no deviation from this in terms of drawdown over the relevant time period. On some occasions, water levels may have exceeded this for a short time during snow melt or heavy rains, but this has no negative consequences for the coregonid fish species.

Dimorphic whitefish systems consisting of a predominantly pelagic DR-whitefish and a mainly epibenthic SR-whitefish are found in numerous lakes in the Finnmark region [21]. Only the Pasvik river system has been affected by an invasion of the congeneric non-native vendace. Research on other dimorphic whitefish systems indicate their stability over time, both in pristine conditions and when subjected to quite dramatic management measures. Survey fishing in the two pristine lakes Lahpojavri and Suohpatjavri demonstrated the temporal stability of the dimorphic systems in terms of both gillraker distribution and relative CPUE in gillnet catches [22]. Survey fishing was performed in 1993 and 2007 in Lahpojavri, and 1996 and 2007 in Suohpatjavri.

In Lake Stuorajavri, the dimorphic whitefish population was subjected to an intensive stock depletion programme in 1981–83, the management goal being to improve fish quality and initiate commercial exploitation [51]. A total of 101 metric tons, or 40.5 kg ha^−1^ of fish was removed from the lake, of which 38.4 kg ha^−1^ was whitefish. This had a dramatic effect on age structure, growth rates, and parasite infection levels [51]. Enhanced exploitation levels were not maintained after 1983, and after 10–15 years of almost zero exploitation, both whitefish morphs had age structures, growth rates and parasite infection levels similar to the pre-depletion period [23].

Thus, it appears that the only known environmental change in Lake Vaggatem which may have brought about the change in the whitefish populations is the invasion of vendace. The apparent stability of similar dimorphic whitefish systems in other lakes in the region supports this conclusion.

Our findings are in accordance with the expectation that the whitefish morph most similar to the invader in trophic morphology, diet and habitat use would be more severely impacted by the invasion. The negative impact of the previous year’s vendace density on the condition of both vendace and DR-whitefish indicated that high vendace density mediate reduced zooplankton resources [13], [52], and thus impoverished living conditions for planktivorous fish. We observed several responses in DR-whitefish supporting this: i) an immediate habitat shift from the pelagic to the epibenthic habitat with a subsequent dietary shift [19], [53]; ii) the subsequent increased annual mortality affecting population density; and iii) the substantial decrease in population density after 4–5 years. This delay in population density response is likely related to the fact that DR-whitefish has a generation time (to age at first maturity) of 3–4 years. Furthermore, iv) the interactions between vendace and DR-whitefish seem to weaken after the first 7–8 years when the population density of DR-whitefish had decreased to a low level. This also makes sense as several life-history parameters of DR-whitefish returned to pre-invasion levels. We interpret this process to signify intense resource competition between vendace and DR-whitefish when vendace was increasing while DR-whitefish was still abundant [13]. As DR-whitefish became substantially less abundant, it seems reasonable to assume that interspecific resource competition became more relaxed. The MARSS interaction analysis revealed negative, although not significant, interaction effects of vendace on the two whitefish eco-species over the full study period from 1991–2010. Intraspecific interactions (density dependence) within vendace and DR-whitefish were, on the other hand, significant. This is another indication that the system has stabilized, with intraspecific competition dominating over interspecific competition.

SR-whitefish maintained its predominantly benthic habitat use, although SR-whitefish population abundance decreased throughout the study period. The rate of population reduction was lower, and the extent of the reduction much less in SR- than in DR-whitefish. This is also in accordance with our expectations since SR-whitefish and vendace have different trophic niches in terms of diet and habitat use [13], [17], [53]. The substantially longer generation time in SR-whitefish (6–7 years) compared to both vendace (2 years) and DR-whitefish (3–4 years) may also mask short term interactions affecting mortality rates. As many age groups are present in the SR-whitefish population, short term variation in the competition level caused by varying densities of vendace may be buffered and not reflected in the population densities of SR-whitefish.

Asymptotic length L_∞_ varied more within the two whitefish morphs through time than within the vendace population, but neither in DR- nor in SR-whitefish did we observe any directional response in L_∞_ to the vendace invasion. However, both DR- and SR-whitefish responded to the vendace invasion by reducing their initial growth rates. In SR-whitefish this was limited to the early transient period, while in DR-whitefish it was seen for a period of 7–8 years. SR-whitefish also exhibited slightly increased annual mortality during the period 1998 to 2006, likely reflecting that this eco-species was influenced by high densities of vendace, either directly or indirectly (i.e., mediated by the niche shift of the DR-whitefish).

The population dynamics and life history parameters for the three coregonid eco-species demonstrated a gradient from r- to K-selected life histories [54]. Egg size is generally correlated with individual juvenile survival probability [55]. In vendace, eggs were small and individual fecundity high relative to body size, although it seems that small body size somehow restricted the proportion of energy channelled into reproduction, as indicated by the low gonadosomatic index (GSI). Vendace matured early and at a small body size, and had a consistently high annual mortality, fluctuating population densities, short generation time and an opportunistic strategy with offspring survival probability traded off by high fecundity. Based on these life history traits vendace can be termed the most r-selected coregonid of the three, exhibiting the most typical opportunistic strategy according to the model of Winemiller and Rose [56]. At the opposite end of the continuum, SR-whitefish matured late at a large body size, and had a long life span with a low annual mortality. All three eco-species generally seem to spawn every year after maturation. The differences in mortality and life expectancy after maturation would cause the number of spawnings per individual to increase from vendace, through DR-whitefish to SR-whitefish. SR-whitefish eggs were large, and a large body size results in high reproductive investment in terms of GSI and fecundity. The SR-whitefish life history is therefore relatively K-selected, following an equilibrium strategy [56] with focus on a high survival rate, a long life span, and stable recruitment. DR-whitefish was intermediate in all life history parameters, i.e. age and size at maturity, mortality rates, egg size, fecundity, and GSI.

Suggestively, these life-history differences are related to the characteristics of the main food sources of the eco-species (cf. [18], [52]). The cladoceran zooplankton prey has a short generation time spanning weeks or months, whereas important littoral prey such as chironomids and molluscs have long generation times from one to several years. Hence, variation and changes in zooplankton prey abundance will occur much faster than in littoral prey abundance. A rapid and opportunistic life history is therefore better matched with zooplankton as a food resource, as compared to the more slowly renewable littoral food resources. The match between hatching of fry from eggs and the zooplankton spring bloom is also crucial [57], [58], and the r-selected vendace will in years with good match be able to successfully translate the zooplankton peak into high numbers of surviving offspring. DR-whitefish is better adapted to the zooplanktivore niche than SR-whitefish both in terms of morphology, ecology and life-history [17], [18], but it is less specialized as compared to the obligate planktivore vendace [19].

The success of vendace as an invader is probably a result of the short and opportunistic life history, combined with the superior ability as a zooplanktivorous competitor [19], [24]. This enabled a swift population increase for vendace in the new environment, but also a population crash, likely due to a temporary exhaustion of the plankton as a food resource [13], [52]. This probably resulted in the unusual observation that fish larvae were included in the diet of vendace [59]. Even after the population crash and subsequent increase in numbers, vendace density has continued to vary considerably from year to year. Large variation in cohort strength appears to be common in vendace populations [34], [36].

It seems that DR-whitefish still utilize resources which were included in its diet before the vendace invasion, but the subsequent changes in the zooplankton community [52] render DR-whitefish less able to utilize this food source. The co-existence of DR-whitefish with the invading vendace is based partly on benthic diet resources that only to a limited extent are exploited by vendace, but which are more efficiently utilized by SR-whitefish [13]. Thus, DR-whitefish seem to be squeezed between the two other eco-species, with a reduction in carrying capacity compared to the situation before the vendace invasion. After the population decrease to the new carrying capacity, it appears that the DR-whitefish is regulated more by density dependence than by interspecific competition, and it may therefore be able to persist in the system.

Vendace in Lake Vaggatem do not grow to a body size which is past the predation window; they remain vulnerable to predation from the gape-limited predators such as brown trout and pike [60] throughout life. The rapid life history with early reproduction and high mortality may be an adaptive response to this. SR-whitefish, on the other hand, grow to sizes which are out of reach of these predators. This difference between vendace and SR-whitefish was clearly reflected in the large difference in annual mortality rates documented in this study. The mortality rates of DR-whitefish were intermediate between the two other eco-species. The mortality of DR-whitefish increased by about 50% from the 1991–93 to the 1995–97 period, reflecting the DR-population decline during the boom-phase of the vendace population. Thereafter DR-whitefish mortality rates have been decreasing, reaching a level similar to the 1991–93 period by 2007–2009. SR-whitefish did also show signs of increased mortality rates, but to a lesser extent than DR-whitefish. The response of SR-whitefish to vendace establishment may also be indirect, mediated through the habitat shift of DR-whitefish to the epibenthic habitat, with a subsequent dietary shift to utilize more benthic prey [19], [28]. Although the total density of DR-whitefish decreased, a higher level of resource competition in the epibenthic zone may be the result of the niche shift by the remaining DR-whitefish to this habitat. Another factor contributing to increased mortality in SR-whitefish could be that reduced DR abundance mediated a shift in pike prey choice from DR-whitefish to medium-sized SR-whitefish. Unfortunately, we do not have long-term pike diet data to investigate this.

The response of the native coregonid fishes to the vendace invasion in Lake Vaggatem was mainly seen in DR-whitefish, which is the eco-species with resource requirements similar to the invader. The response of SR-whitefish, on the other hand, was much less apparent. This eco-species is ecologically more different from vendace. Phylogenetically, the two whitefish forms are at the same distance from vendace. The significance of ecological similarity in predicting the impact of an invasion is also seen in other cases of fish introductions. When whitefish is introduced in lakes with Arctic charr (*Salvelinus alpinus* (L.)), which has similar ecology, but is less efficient as a zooplanktivore, Arctic charr shows a dramatic decline in population numbers, and the remaining fish are relegated to the less profitable profundal habitat [61], [62], [63]. It should be noted that whitefish and Arctic charr, although both in the Salmonidae family, are more phylogenetically distant than whitefish and vendace, which are con-generic. In shallow lakes, i.e. with no alternative habitats available, Arctic charr may become extirpated [64]. Direct competition for resources is obviously important for species interactions (cf. [19]), and we may argue that the phylogenetic distance between native species and an invader is of less importance than their ecological similarity. Thus, it may seem that the naturalization hypothesis *per se* offer little predictive power about invasion effects.

In conclusion our data illustrate an ecological drama during 20 years since the appearance of vendace, with strong direct competition, leading to reduction, but not extinction, of the taxonomically related and native DR-whitefish competitor. Indirect effects are also demonstrated in the ecologically less similar SR-whitefish, showing damped system interactions with more complex causes.
